# Fundamentals of Microbial Community Resistance and Resilience

**DOI:** 10.3389/fmicb.2012.00417

**Published:** 2012-12-19

**Authors:** Ashley Shade, Hannes Peter, Steven D. Allison, Didier L. Baho, Mercè Berga, Helmut Bürgmann, David H. Huber, Silke Langenheder, Jay T. Lennon, Jennifer B. H. Martiny, Kristin L. Matulich, Thomas M. Schmidt, Jo Handelsman

**Affiliations:** ^1^Department of Molecular, Cellular and Developmental Biology, Yale UniversityNew Haven, CT, USA; ^2^Institute of Ecology/Limnology, University of InnsbruckInnsbruck, Austria; ^3^Department of Ecology and Evolutionary Biology, University of CaliforniaIrvine, CA, USA; ^4^Department of Aquatic Sciences and Assessment, Swedish University of Agricultural SciencesUppsala, Sweden; ^5^Department of Ecology and Genetics/Limnology, Uppsala UniversityUppsala, Sweden; ^6^Department of Surface Waters – Research and Management, Eawag: Swiss Federal Institute of Aquatic Science and TechnologyKastanienbaum, Switzerland; ^7^Department of Biology and Gus R. Douglass Institute, West Virginia State UniversityDunbar, WV, USA; ^8^Department of Biology, Indiana UniversityBloomington, IN, USA; ^9^Department of Microbiology and Molecular Genetics, Michigan State UniversityLansing, MI, USA

**Keywords:** microbial ecology, disturbance, stability, sensitivity, structure-function, perturbation, community structure, time series

## Abstract

Microbial communities are at the heart of all ecosystems, and yet microbial community behavior in disturbed environments remains difficult to measure and predict. Understanding the drivers of microbial community stability, including resistance (insensitivity to disturbance) and resilience (the rate of recovery after disturbance) is important for predicting community response to disturbance. Here, we provide an overview of the concepts of stability that are relevant for microbial communities. First, we highlight insights from ecology that are useful for defining and measuring stability. To determine whether general disturbance responses exist for microbial communities, we next examine representative studies from the literature that investigated community responses to press (long-term) and pulse (short-term) disturbances in a variety of habitats. Then we discuss the biological features of individual microorganisms, of microbial populations, and of microbial communities that may govern overall community stability. We conclude with thoughts about the unique insights that systems perspectives – informed by meta-omics data – may provide about microbial community stability.

## Introduction

In habitats as diverse as soil and the human body, key ecosystem processes are driven by microbial communities – local assemblages of microorganisms that interact with each other and their environment (Konopka, 2009). Thus, microbiology research in biomedical, environmental, agricultural, and bioenergy contexts shares a common challenge: to predict how functions and composition of microbial communities respond to disturbances (Robinson et al., 2010a; Gonzalez et al., 2011a,b; Costello et al., 2012).

Here, we introduce a breadth of topics that provide insight into the responses of microbial communities to disturbance. We first highlight key concepts from ecology that are useful in thinking about microbial stability, pointing readers to an extensive literature on the subject of disturbance and community stability. We then summarize the current state of knowledge about resistance and resilience of microbial communities inhabiting a variety of ecosystems, emphasizing overarching trends gleaned from a review of 247 representative studies. We next provide a synthesis of the properties of individual microorganisms, populations, and communities that influence microbial community stability. Finally, we discuss insights into stability that may emerge from a systems-level perspective – describing microbial communities as networks of genes, transcripts, proteins, and metabolite signals.

## Key Concepts from Ecology

### Defining disturbance

Disturbances are causal events that either (1) alter the immediate environment and have possible repercussions for a community or (2) directly alter a community (Rykiel, 1985; Glasby and Underwood, 1996). After disturbance, community members may die (mortality) or change in their relative abundances (Rykiel, 1985). A disturbance can be difficult to define, as its definition depends on scale and context. Disturbances occur at various spatial and temporal scales (Paine et al., 1998) with different frequencies (number of occurrences per unit time), intensities (magnitude of the disturbance), extents (proportion of the ecosystem affected), and periodicities (regularity of occurrences; Grimm and Wissel, 1997). Disturbances may also be defined relative to the disturbance regime of an ecosystem, such as a fire or flooding cycle.

**Figure 1:**
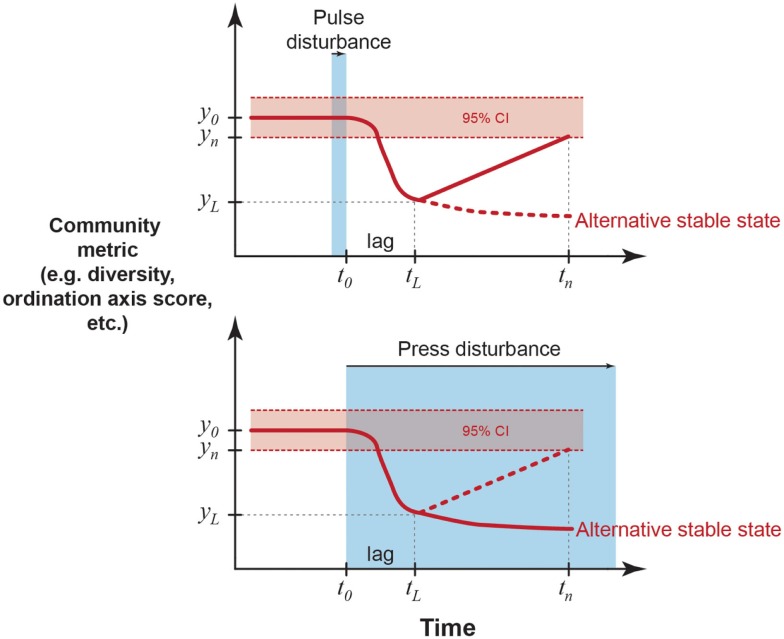
**Examples of quantitative definitions of resistance and resilience from ecology (Westman, 1978; Orwin and Wardle, 2004; Suding et al., 2004)**. A microbial community parameter of interest has a mean value of *y*_0_ and temporal variance, illustrated here by a 95% confidence interval around the mean (though other quantifications of variance, such as standard deviation or variance ratios may be used). A pulse disturbance ends (or a press disturbance begins) at time *t*_0_ and the parameter changes by |*y*_0_ − *y_L_*| after a time lag *t_L_* − *t*_0_. Resistance (RS) is an index of the magnitude of this change.
(1)$$RS=1-\frac{2\left|y_{0}-y_{L}\right|}{y_{0}+\left|y_{0}-y_{L}\right|}$$
Resilience (RL) is an index of the rate of return to *y*_0_ after the lag period,
(2)$$RL=\left[\frac{2\left|y_{0}-y_{L}\right|}{\left|y_{0}-y_{L}\right|+\left|y_{0}-y_{n}\right|}-1\right]÷\left(t_{n}-t_{L}\right)$$
where *y_n_* is the parameter value at measurement time *t_n_*. A parameter is “recovered” when it is statistically indistinguishable from the pre-disturbance mean. Alternatively, the parameter may not recover and instead may stabilize at a new mean value representing an alternative stable state. This possibility is more likely in response to a press disturbance. Further, RS and RL could be related to normalized parameters describing the disturbance (e.g., intensity, duration, frequency of the stressor in relation to the pre-disturbance mean and variance), which is useful for cross-system comparisons.

Disturbances are often classified as *pulses* or *presses* depending on their duration (Bender et al., 1984). In general, pulse disturbances are relatively discrete, short-term events, whereas presses are long-term or continuous (Figure 1). However, these time scales may differ depending on the generation time of the community of interest. For instance, a tree falling in a forest may create a press disturbance to the underlying soil microorganisms, whereas the same event might be considered a pulse disturbance to nearby understory vegetation. Though the distinction between pulse and press disturbances has received much attention in the ecology literature, there is less discussion of patterns of microbial community responses to pulses and presses. However, microbial community responses to pulses and presses are important to consider in the context of global climate change. With global changes, pulse disturbances (e.g., extreme weather events) are expected to increase in frequency, and ongoing press disturbances are expected to continue (e.g., atmospheric increases in carbon dioxide, ocean acidification; Intergovernmental Panel on Climate Change, 2007). Therefore, throughout this work, we discuss microbial community responses in both pulse and press disturbance scenarios.

### Defining stability

Disturbance and community stability are necessarily related, as stability is defined as a community’s response to disturbance (Rykiel, 1985). Here, we adopt definitions most similar to Pimm (1984), in which stability is comprised of resistance and resilience (Table 1), two quantifiable metrics that are useful for comparing community disturbance responses and have precedent in the microbial ecology literature (e.g., Allison and Martiny, 2008). However, readers should be aware that the ecology literature includes many definitions of stability, and a full examination of these definitions is available elsewhere (Grimm and Wissel, 1997). Here, resistance is defined as the degree to which a community is insensitive to a disturbance, and resilience is the rate at which a community returns to a pre-disturbance condition (Pimm, 1984). A related concept, sensitivity, is the inverse of resistance and defined as the degree of community change following a disturbance. Both resistance and resilience are usually quantified in relation to a community’s level of intrinsic variability, sometimes referred to as the “normal operating range” (van Straalen, 2002). There are many methods in the literature [see a recent summary by Griffiths and Philippot (2012)] for comparing resistance or resilience across communities (Orwin and Wardle, 2004).

**Table 1:** **Common terms for disturbances, community responses, and community outcomes**.

**DISTURBANCE TERMS**	
Disturbance	A causal event that causes a discrete change in the physical or chemical environment that has anticipated effects on a community (Rykiel, 1985; Glasby and Underwood, 1996)
Press disturbance	A continuous disturbance that may arise sharply but reaches a constant level that is maintained over a long period of time (Lake, 2000)
Pulse disturbance	A short-term, often intense disturbance that rapidly decreases in severity over a short period of time (Lake, 2000)
**COMMUNITY TERMS**	
Community	An assemblage of microorganisms that live in the same locality and potentially interact with each other or with the environment (Konopka, 2009)
Metacommunity	Within a regional landscape, a set of local communities whose members are linked by dispersal (Wilson, 1992; Logue et al., 2011)
**COMMUNITY RESPONSE TERMS**	
Stability	The tendency of a community to return to a mean condition after a disturbance (Pimm, 1984); includes the components of resistance and resilience
	Ecological stability can be measured in many ways, including the persistence of populations through time, constancy of ecological attributes through time, resistance to a disturbance, or resilience after a disturbance (Worm and Duffy, 2003)
Resistance	The degree to which a community withstands change in the face of disturbance (Pimm, 1984; Allison and Martiny, 2008). Inverse of *sensitivity*
Sensitivity	The degree to which a community changes in response to disturbance, the inverse of resistance
Resilience	The rate at which a microbial community returns to its original composition after being disturbed (Allison and Martiny, 2008). Commonly referred to as community recovery. Inverse of return time
**COMMUNITY OUTCOMES**	
Stable state	A condition where a community returns to its original composition or function following a disturbance (Beisner et al., 2003). Also known as community equilibrium or an attractor
Alternative stable state	A condition where a community moves to a different but stable composition or function following a disturbance. One of multiple, non-transitory stable states in which a community can exist (Beisner et al., 2003)
Regime shift	A large change in community composition arising from a shift between alternative stable states (Scheffer and Carpenter, 2003)

A community’s stability can be investigated in terms of functional or compositional parameters. In microbial ecology, many studies also focus on the degree to which functional and compositional stability are related. This may depend in large part on the particular function of interest (Schimel, 1995). For functions that are carried out by many taxa (Schimel, 1995), i.e., communities harboring a high degree of functional redundancy, changes in community composition may not correspond with changes in functional rates (Allison and Martiny, 2008). Alternatively, for functions performed by only a few taxa (for example, in situations of ecological coherence of closely related taxa, Philippot et al., 2010), the sensitivity and resilience of this function may closely follow changes in the abundance of those taxa. Notably, estimates of resistance and resilience for the same microbial community may have different values depending on whether compositional or functional responses are measured and on which functions are used to assess stability.

**Figure 2:**
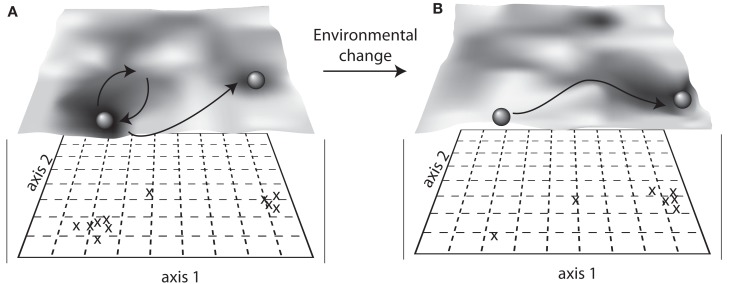
**Alternative equilibria, also called alternative stable states, visualized with a stability landscape**. Here, changes in community composition are assessed using axis scores from an ordination (e.g., principal coordinates analysis (PCoA) of Bray–Curtis similarities) before **(A)** and after **(B)** environmental change. The overlay “terrain” of the landscape shows the different stable states as basins, and the community is represented as a ball that is either maintained in its original basin or displaced to a new basin after a disturbance. Community resilience is represented by the slope of the basin walls, showing a rate of return to the original stable state.

One perspective of stability, sometimes referred to as “ecological resilience,” relies on the existence of many stable states in which a community may reside (e.g., Holling, 1973, 1996; Botton et al., 2006). For instance, a community may shift to a new stable state when subjected to a press disturbance (Figure 1). The existence of multiple equilibria can also be illustrated by the concept of a stability landscape (Beisner et al., 2003; Scheffer and Carpenter, 2003; Collie et al., 2004; Folke et al., 2004), which can be used to conceptualize microbial community responses to disturbance (Blodau and Knorr, 2006; Mao-Jones et al., 2010; Bürgmann et al., 2011; Seto and Iwasa, 2011). In a visualization of a stability landscape, a ball represents a community that can exist in one of many different equilibrium states (basins) within the stability landscape (Beisner et al., 2003; Scheffer and Carpenter, 2003; Figure 2). A disturbance event is analogous to applying a force to the ball within its basin. A community may resist the disturbance, which is represented by the ball remaining in its basin. Alternatively, the community could change but exhibit resilience, which is represented by the ball moving outward from the basin but then returning to its original location (Figure 2A). If resistance and resilience are low or the disturbance strong enough, the community may shift to an alternative equilibrium (also called alternative stable state), represented by the ball moving into a new basin. Once in an alternative equilibrium, the community’s return to the previous composition or function may be difficult (Botton et al., 2006). Moreover, environmental conditions shape the stability landscape (Figure 2B). Thus, if a press disturbance permanently alters the stability landscape, this will have implications for community stability and the likelihood of community shifts to alternative stable states.

Studies of alternative stable states and regime shifts in microbial systems remain rare (Botton et al., 2006), though the conceptual framework is gaining popularity, especially among researchers interested in the human gut microbiome (Lozupone et al., 2012), as the existence of alternative stable states may provide explanation as to the immense variability observed within and among individual gut microbial communities. There is also evidence of alternative stable states in the vaginal microbiome, where eight “super-groups” of distinct microbial assemblages have been detected across hundreds of healthy women (Zhou et al., 2007). Additionally, there are a few concrete examples of microbial communities that exhibited regime shifts. For instance, increased influx of groundwater triggered a functional regime shift from iron-reduction to sulfate-reduction in anoxic sediments of mine drainage lakes (Blodau and Knorr, 2006), and operational changes triggered a compositional and functional regime shift in a sequencing batch reactor for nitrogen elimination from urine (Bürgmann et al., 2011). Furthermore, regime shifts in microbial communities may have far-reaching consequences for ecosystems, as suggested by theoretical models of coral reef microbial communities that shift composition from antibiotic-producers to pathogens (Mao-Jones et al., 2010). As time series studies are extended to include more disturbance events, alternative stable states may be detected for other microbial communities. In the example in Figure 2, microbial community composition and function are mapped using multivariate ordination to visualize a stability landscape.

### Measuring stability: Community response to disturbance

Ecologists have long considered how to quantify resistance and resilience of communities and their functions. One experimental design that specifically addresses the impact of a disturbance is “before-after-control-impact” (BACI; Stewart-Oaten et al., 1992; Smith et al., 1993; Ellis and Schneider, 1997; Stewart-Oaten and Bence, 2001; Fraterrigo and Rusak, 2008). But, BACI has known limitations [discussed in Underwood (1994)], including violation of assumptions of non-independence of samples collected over time. Thus, multivariate autoregressive moving-average models, which remove temporal autocorrelation, have been applied to understand the contribution of species and environmental interactions to the stability of communities (e.g., Ives et al., 2003). Additionally, Bayesian approaches such as dynamic linear models (DLM) account for the autocorrelation of time series data, and estimate the sensitivity of data to disturbance (e.g., Carpenter and Brock, 2006). Techniques such as DLM also make projections about future behavior of a response variable based on pre-disturbance data distribution (sometimes called intervention). There are many other techniques that quantify temporal variability to assess the impact of disturbance and measure resilience (e.g., Underwood, 1994; Ives, 1995; Ives et al., 2000; Fraterrigo and Rusak, 2008). Methods for handling temporal datasets (e.g., Lennon, 2011) will become increasingly useful to microbiologists as more and longer time series of microbial communities become available. However, the species-rich nature of many microbial datasets, especially those generated using high-throughput sequencing, present computational challenges that will likely require new methods of statistical analysis (Gonzalez et al., 2011a).

When measuring stability, it is important to distinguish between responses to pulse and press disturbances, as recovery may be quantified by slightly different methods (Glasby and Underwood, 1996). Ideally, resilience to a press disturbance should be determined after the community composition or function reaches its maximum deviation from the expected mean (Figure 1). With a press, there is often more uncertainty about when the disturbance has caused the maximal change in the community, especially if it is unknown when the press disturbance began and whether it has ceased. Therefore, when to establish the baseline for measuring resilience after a press disturbance is less obvious, and therefore a major research challenge. By contrast, response to a pulse can be defined immediately after the pulse ends, although there may be a time lag before the disturbance response is completed.

Additionally, experimental settings allow pulse and press responses to be compared directly and described relative to one another. For example, tropical soil microbial communities exposed to fluctuating (pulse) anoxic-to-oxygenic conditions were compared to those exposed to continuous (press) anoxic or oxygenic conditions (DeAngelis et al., 2010). This pulse-press comparison revealed that communities exposed to repeated redox fluctuations were more diverse and more active (assessed by their RNA to DNA ratio) than communities exposed to a constant condition. Furthermore, when microbial communities are monitored long-term, pulse and press responses also may be compared *post hoc*. In these cases, community stability could serve as an indicator of unmeasured or unobserved pulse and press disturbances: short-term variability around a baseline (equilibrium) may be a sign of pulses while gradual shifts in the baseline may be a sign of presses (Shade et al., 2012).

### Community invasibility as an indicator of stability

Invasion, the successful establishment of a non-native organism in a community (Litchman, 2010), can provide an indicator for both compositional and functional stability. Invasion is unique in that it can be considered both a cause and consequence of disturbance. In studies of communities with larger organisms, a well-known consequence of community disturbance is reduced resistance to invasion by alien species (called “niche opportunity”; Shea and Chesson, 2002), but its parallel use as a functional indicator of stability in microbial communities has been limited, with a few exceptions (e.g., Robinson et al., 2010b). Community invasion does, however, have a long history in microbial ecology in the context of agricultural inoculants and veterinary and clinical probiotics. For example, a century ago, Ilya Metchnikoff explored invasion of his own gut microbiome by lactobacilli consumed in sour milk, and found that the lactobacilli did not invade his gut community and needed to be replenished frequently to obtain the salubrious effects on his health that he reported (Metchnikoff, 1908; Schmalstieg and Goldman, 2008). A century of study of probiotics revealed the challenge of establishing new strains of bacteria in the mammalian gut, with many studies documenting disappearance of introduced strains within hours of entrance into the gastrointestinal tracts of pigs (e.g., Gardiner et al., 2004) and humans (Robins-Browne and Levine, 1981; Ventura and Perozzi, 2011). These studies contributed to the broadly held sense that microbial communities are resistant to invasion, embodied in the concept of “colonization resistance” of the human microbiome (Savage, 1977; Hopkins and Macfarlane, 2003; Johnson-Henry et al., 2008; Britton and Young, 2012).

## Knowns and Unknowns about Microbial Community Stability: An Updated Investigation of the Literature Considering Responses to Pulse and Press Disturbances

Recent studies have reported that, in general, soil microbial communities are not resistant to disturbances, as measured by composition, and that even within several years many communities fail to recover entirely (Allison and Martiny, 2008). To extend this analysis to non-soil communities, we explored the literature for studies investigating microbial community stability in the face of disturbance (see Appendix). We considered 247 studies, and these studies included a total of 378 investigations of soil, marine and freshwater, engineered (e.g., wastewater treatment, bioreactors), and host-associated (gut) systems to discern patterns of stability that may be broadly applicable to microbial communities. From this exploration, we chose representative examples from the literature to illustrate key points, as our search was not intended to be exhaustive. We focused our comparisons on microbial community responses to pulse and press disturbances.

**Figure 3:**
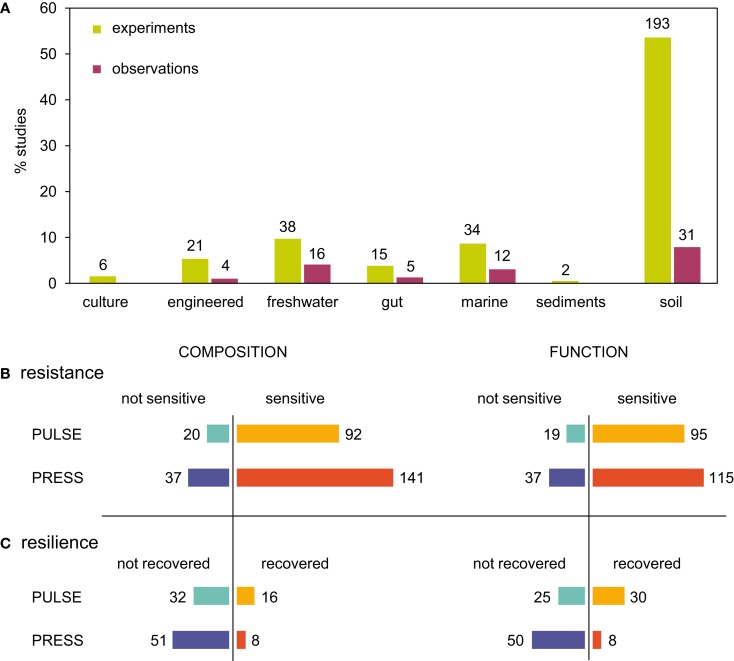
**Summary of a literature survey of microbial community responses to pulse and press disturbances**. The survey included studies that investigated changes in microbial community structure after biological, chemical, or physical disturbances. **(A)** Representation of investigations across ecosystem types, and by whether the investigation was a designed experiment or opportune *in situ* observations after a disturbance. There were 378 total investigations from 247 total studies, as some studies investigated more than one disturbance or measured more than one function, and some studies did not report either. **(B)** Resistance was determined by sensitivity (change in composition or function after disturbance). Some investigations measured both composition and function, and were included in both charts. **(C)** If a community was sensitive to disturbance, resilience was measured as recovery to pre-disturbance composition or function. Many investigations that reported community sensitivity did not assess recovery.

Investigations of microbial community stability generally fell into two broad classes: *observations*, which typically involved *in situ*, large-scale disturbances (e.g., deforestation, typhoons, temperature changes), and *designed experiments* that usually involved small-scale disturbances (e.g., nutrient amendment, temperature alterations, or fumigation, Figure 3A). Resistance and resilience were assessed either based on microbial community composition or function (Allison and Martiny, 2008; Little et al., 2008), which are sometimes linked (Balvanera et al., 2006; Cardinale et al., 2006). Many studies (23%) measured only microbial composition, assessed by multivariate analysis of molecular fingerprints or 16S rRNA gene sequences, as the response variable to assess stability. Some studies (18%) measured community functions (respiration, biomass production, or activity of extracellular enzymes). Finally, a large number of studies (58%) measured both community composition and function.

**Figure A1:**
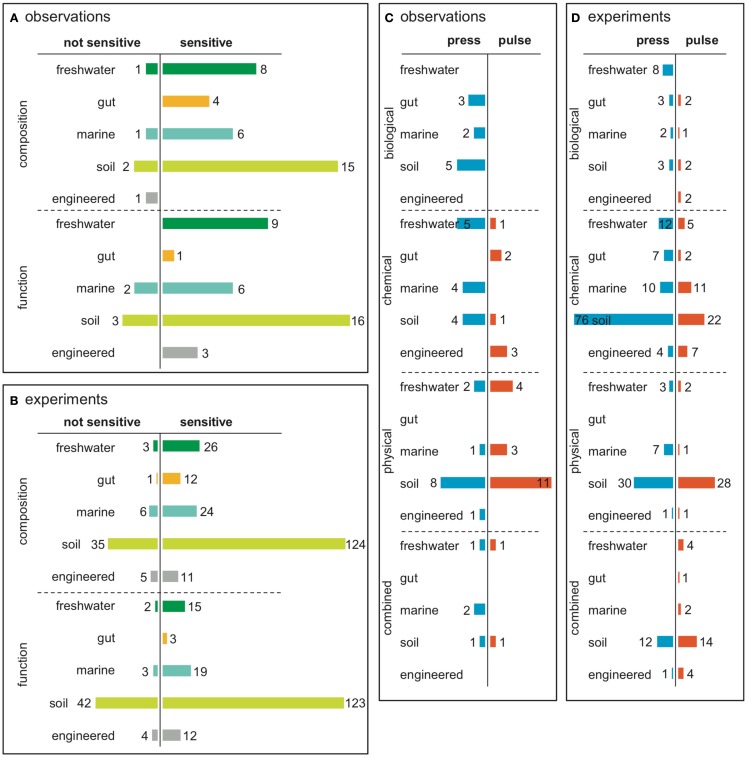
**Summary of the literature survey of microbial community sensitivity to disturbances, grouped by habitat**. There were 378 total investigations gleaned from 247 total studies, as some studies investigated more than one disturbance or measured more than one function. Note that a few studies that considered uncommonly investigated habitats (one wetland, two sediment, six culture-based, and one leaf/detritus) are not shown in this figure. Investigations were classified as either **(A–C)** observations or **(B–D)** experiments. **(A,B)** shows the sensitivity of microbial communities by observations and experiments, and **(C,D)** shows the distribution of different disturbance types between press and pulse disturbance studies. Of the investigations included, 220 investigations reported press disturbances, and 148 were pulses, four did not report press or pulse (NA), and six reported combined press and pulse. Note the lack of observational studies investigating pulsed biological disturbance (e.g., blooms) and the general shortage of work on combined effects of biological, chemical, and physical disturbance.

From our examination of 310 experimental and 68 observational investigations of microbial responses to disturbances, 82% reported sensitivity to disturbance, either in composition (26%), function (21%), or both (35%, Figure 3B). This is in agreement with previous findings for soil communities (Allison and Martiny, 2008), and suggests that most microbial communities may be sensitive to disturbances. One caveat to this finding is that it may be more difficult to publish results of experiments in which communities did not change when challenged with a disturbance, and so this finding may reflect a potential bias in the literature. A habitat-by-habitat summary of sensitivity to disturbance is given in Figures A1A,B in Appendix. Though we considered microbial communities from many habitats, soil communities were most represented, affirming that the majority of disturbance investigations in microbial ecology are from soil habitats.

**Figure A2:**
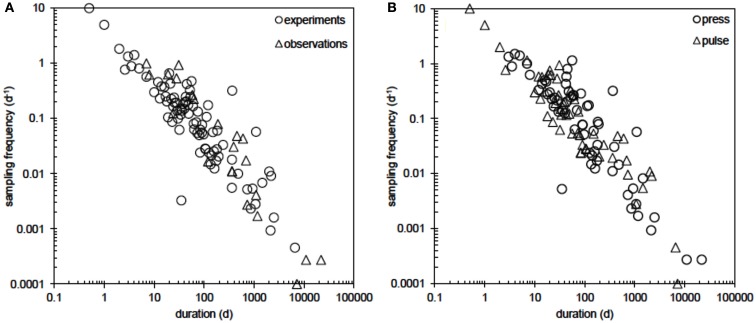
**The impact of sampling frequency and duration on our knowledge of microbial disturbance responses in observational and experimental studies (A) and in studies applying press and pulse disturbances (B)**. Experiments apparently well cover the temporal scales of disturbances anticipated by observational studies **(A)** and surprisingly there is no pronounced bias toward short-term studies with a high sampling frequency in pulse disturbance studies **(B)**. However, the relationship between study duration and sampling frequency **(A,B)** indicates logistic and/or conceptual constraints, which may limit our ability to predict microbial community responses to disturbance. “d” is days.

Only a few investigations explicitly measured resilience (Figure 3C). Of those 148 investigations that reported community sensitivity to disturbance and also examined recovery, only a small fraction reported return to pre-disturbance composition (13%), function (8%), or both (2%). However, it was unclear whether resilience was not observed in some investigations because of biases in sampling intensity or duration after the disturbance (Figure A2 in Appendix) or because the communities were not, in fact, resilient. The normal variability of microbial communities in the absence of any disturbance event was also often unreported. Without *a priori* knowledge of community turnover, it may be difficult to inform post-disturbance sampling intensity or duration. Thus, knowledge of baseline microbial community stability as well as post-disturbance dynamics remains limited for many habitats and contexts.

Our results suggest that microbial communities are equally sensitive to pulse and press disturbances (Figure 3B). Drawing on the subset of investigations that assessed resilience, our results hint that microbial communities may be more resilient after pulse disturbances than after press disturbances (Figure 3C). Recovery from pulse disturbances was reported more often for microbial community function than for composition, while recovery from press disturbances was approximately the same for both function and composition. As more disturbance studies become available, further work will be needed to compare resilience quantitatively across press and pulse disturbances, as different disturbance types (chemical, biological, physical, combination) were differently represented within pulse and press investigations (Figures A1C,D in Appendix). For example, though physical disturbance types were represented approximately equally in both pulse and press investigations, press disturbances included a larger representation of chemical disturbance types than pulse.

Additionally, the literature survey results draw attention to our current knowledge gaps regarding microbial community responses to pulse and press disturbances. Specifically, there is limited understanding of microbial responses to biological pulse disturbances and to disturbance combinations for both pulses and presses. First, investigations of microbial responses to biological disturbance types were rare, especially in pulse disturbance scenarios (Figures A1C,D in Appendix). Examples of pulse biological disturbances include phytoplankton blooms, which not only impact neighboring microbial communities but also have implications for both heterotrophic and autotrophic contributions to global carbon cycling (e.g., Teeling et al., 2012). Therefore, understanding pulse biological disturbances remains an important gap to fill. Second, investigations of disturbance combinations were also uncommon among the literature surveyed, though pulse disturbances included a larger representation of disturbance combinations than press (Figures A1C,D in Appendix). Compounded disturbances include those that occur simultaneously or within the recovery time of a preceding disturbance. Because compounded disturbances may lead to regime shifts (e.g., Paine et al., 1998), studying microbial community responses to compounded disturbances is increasingly important in face of global climate change.

It is interesting that though many studies measured functional responses (63%), there has been limited conceptual development on the role of pulse and press disturbances in driving relationships between microbial community composition and function. The presence of functionally redundant species in microbial communities has been suggested to increase functional resilience (e.g., Allison and Martiny, 2008); however, the degree of functional redundancy among microorganisms remains controversial. Diversity – function relationships could be probed by asking whether press and pulse disturbances select for different community memberships (see Biological Features That Contribute to Microbial Resistance and Resilience). Applying combinations of press and pulse disturbances to microbial communities could create gradients in community diversity that may clarify the role of disturbance in driving diversity-function relationships.

The results of our literature survey reveal that we have much to learn about the nature of change and recovery for microbial communities from many habitats. Conceptual progress across disciplinary boundaries within microbial ecology could be achieved by cross-system comparison of stability. However, we currently lack a common framework and standard format of reporting compositional and functional responses to disturbance, which inhibits more quantitative cross-system comparisons. There has been recent progress to standardize microbial community data in the new biological observation matrix (.biom taxa table; see biom-format.org), as used by concerted efforts to collect and curate large microbial datasets, such as by the Earth Microbiome Project (Gilbert et al., 2010). This and similar efforts will support development of disturbance theory for microbial ecology.

## Biological Features that Contribute to Microbial Resistance and Resilience

**Figure 4:**
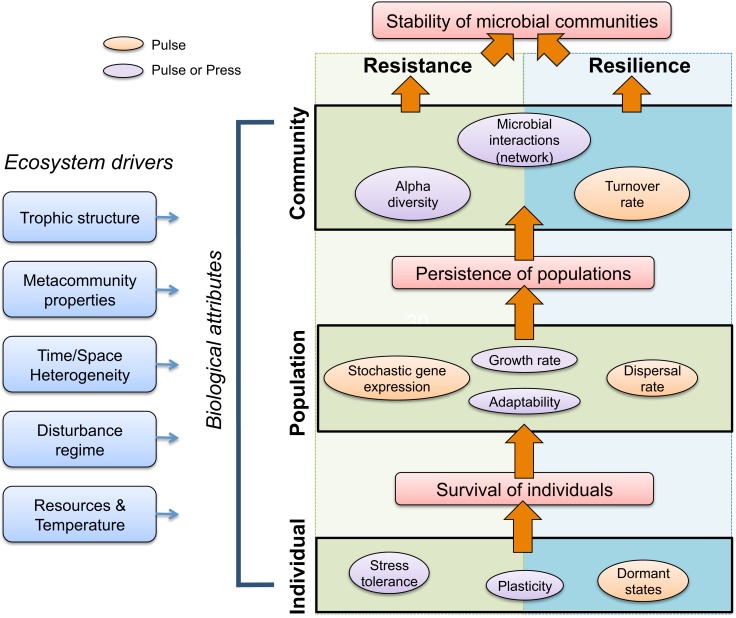
**Conceptual model of biological and ecosystem properties governing microbial community resistance to and resilience after disturbance**. Stability of microbial communities in the face of disturbances is influenced by individual-, population-, and community-level biological attributes that contribute to community resistance (left, green background) and/or resilience (right, blue background), or both (center). Individuals withstand or survive disturbances and promote persistence of populations, which in turn promote overall community stability (orange arrows). Ecosystem drivers (leftmost blue boxes), such as trophic structure and disturbance regime, shape biological attributes, and also contribute to resistance and resilience. Finally, we hypothesize that biological attributes will be differently advantageous given a pulse (orange or purple) or press (purple) disturbance.

Survival of individual cells is a prerequisite for population-level persistence, which is a prerequisite for community-level recovery (Figure 4). In this section, we explicitly focus on compositional, taxon-based resistance and resilience, but make connections to functional resistance and resilience where possible. We hypothesize that there are biological attributes that are of greater importance for microbial community resistance and resilience under pulse disturbance scenarios (orange circles, Figure 4), while other attributes are generally important for both pulse and press disturbances (purple circles).

### Individual properties: Plasticity, stress tolerance, and dormancy

#### Plasticity and stress response

Resistance to compositional change in the face of disturbances is enhanced if a microbial community contains many individuals that have versatile physiologies, or physiological plasticity (Evans and Hofmann, 2012). Bacteria often navigate environmental change by expressing a range of metabolic capabilities (e.g., Meyer et al., 2004; Swingley et al., 2007), and therefore the existing community can confront new conditions through gene expression by individual cells. From an evolutionary standpoint, adaptive gene expression refers to natural selection acting on gene expression (Whitehead and Crawford, 2006), and this phenomenon has been observed, for example, in a yeast model of experimental evolution (Ferea et al., 1999). Furthermore, mixotrophy, or the ability to use many different carbon and energy sources, may be a common phenomenon in natural microbial communities (Eiler, 2006), and provides further support for the notion of individual flexibility in fluctuating environments. Cellular stress responses also provide protection for individual cells from damaging physical factors such as reactive oxygen species, temperature, and ultraviolet light (e.g., Craig, 1985; Ziegelhoffer and Donohue, 2009; Kolowrat et al., 2010). Stress protection in *E. coli* is associated with a progressive decrease in nutritional competence, or the breadth and range of carbon and nutrient resources that a cell can use (Ferenci, 2005; Ferenci and Spira, 2007), which ultimately may reduce the population’s ability to confront other environmental changes. Therefore, there may be fitness costs to microbial stress responses that are manifested in seemingly unrelated metabolic pathways, or costs of maximizing protection against one stress over another. Stress tolerance can contribute to microbial community resistance to pulse or press disturbances, but will depend on the intensity and duration of the disturbance relative to an individual’s levels of stress tolerance (Figure 4).

Compositional stability is mediated, in part, by the ability of individual microorganisms to respond to, accommodate, and exploit environmental change. This highlights a difference between microbial communities and communities of larger organisms: prokaryotes have a degree of physiological plasticity that is unparalleled in the eukaryotic world. The unique ability to shift to an entirely different lifestyle within a short time (as in the classic example of *Rhodobacter sphaeroides*, which can grow anaerobically as a phototroph but also grow aerobically as a chemoheterotroph) is likely to increase the compositional stability while simultaneously reducing the functional stability. Current ecological theory based on plants and animals may not accommodate the enormous physiological plasticity of prokaryotes, which may necessitate development of new principles applicable to microbial communities. We hypothesize that physiological plasticity can contribute to a microbial community’s resistance and resilience to either pulse or press disturbances. But, similar to stress tolerance, the contribution of plasticity to community stability will depend on the intensity and duration of the disturbance relative to an individual’s physiological response.

#### Dormancy

Dormancy is a bet-hedging strategy that allows organisms to enter a reduced state of metabolic activity (Jones and Lennon, 2010); see Lennon and Jones, 2011 for a recent review). A substantial fraction of community members may be dormant or inactive at any given moment (e.g., Pedrós-Alío, 2006; Jones and Lennon, 2010). Dormancy strategies may be common among communities living in temporally dynamic environments, promoting overall compositional stability in fluctuating conditions. Furthermore, the proportion of inactive taxa (and, inactive individuals within a taxon) may signify important environmental differences among communities from similar habitats, as shown, for example, in gut microbial communities from humans with and without irritable bowel syndrome (Rehman et al., 2010).

Dormancy has most likely evolved across the tree of life as a means for contending with temporarily variable environments. It is an advantageous strategy under unpredictable conditions because it allows individuals to maximize their long-term, geometric fitness (de Jong et al., 2011). Recent studies suggest that, in a wide range of ecosystems, a substantial fraction of microbial communities may be metabolically inactive (Lennon and Jones, 2011). This observation has important implications for the resistance and resilience of microbial communities. First, on a population-level, dormancy may prevent the extinction of certain taxa from a system. For example, active individuals of *E. coli* succumb when exposed to certain antibiotics. However, the population can ultimately be rescued, not necessarily by the survival of mutants, but rather by subpopulations of dormant persister cells that become reactivated when antibiotic effects are attenuated (Lewis, 2006). Evidence suggests that microorganisms may use dormancy in a variety of other situations as well, for example when challenged by unfavorable temperatures (Whitesides and Oliver, 1997), starvation (del Giorgio and Gasol, 2008), or predator-induced mortality (Pearl et al., 2008). Second, dormancy has the potential to affect the stability of communities and ecosystem processes. Dormant individuals can be long-lived and contribute to seed banks. It is well documented that seed banks can maintain species diversity (Chesson, 2000), and this diversity may directly contribute to stability of microbial communities via niche complementation and/or functional redundancy (Loreau and Hector, 2001; Petchey and Gaston, 2002). Seed banks may also aid in the recovery of microbial communities from severe disturbance events. For example, dormant seeds often contribute to the reestablishment of plant communities following fire, flooding, or wind storms (Bond and Midgley, 2001). In many cases, microbial communities show similar signs of rapid recovery following catastrophic disturbances (Jones et al., 2008; Shade et al., 2012). Although dormancy may contribute to the stability of these communities, this remains to be tested.

We propose that dormancy is more important for maintaining community stability under pulse disturbance scenarios (Figure 4), as dormancy would be advantageous in a temporarily disturbed ecosystem, but likely less so in a continuously disturbed ecosystem. The exception to this would be a long-lived seed bank of dormant cells that is sustained beyond the effects of a press disturbance. Also, depending on the specific ecosystem changes caused by a press disturbance, environmental cues for “waking” from dormancy (Epstein, 2009) may be altered or absent if a disturbance is continuous.

### Population properties: Adaptation, growth rate, stochastic expression, and dispersal

#### Evolutionary adaptation

Microorganisms generally feature rapid growth, high population densities, and high mutation rates and are capable of recombination via lateral gene transfer, which facilitates response to disturbance events (e.g., Lenski and Bennett, 1993). As such, disturbance often can provide selection pressure that drives diversification (Travisano and Rainey, 1998; Cohan, 2002). A number of studies have shown that rapid evolution of population traits can influence the temporal dynamics of microbial communities. For example, microbial predator-prey dynamics are strongly affected by selection (Yoshida et al., 2003), especially for traits that provide defense against predators (Little and Currie, 2008). Also, rapid evolution can counterbalance the top-down effects of a novel predator on nutrient cycling. For example, viruses introduced into continuous cultures of the picocyanobacterium *Synechococcus* dramatically reduced population densities, thereby increasing availability of the growth-limiting resource, phosphorus. However, *Synechococcus* population densities rebounded with the growth of a virus-resistant *Synechococcus* mutant. Host resistance coincided with reduced nutrient availability (Lennon and Martiny, 2008). Together these studies suggest that rapid evolution is an important mechanism of compositional and functional resistance and resilience to certain disturbances, and is particularly important for response to press disturbances (Cohan, 2002). By contrast, there may be advantages of bet-hedging strategies, such as phenotypic plasticity or dormancy, to contend with disturbance in systems that experience transient pulse disturbances. Adaptability is likely important for microbial community resistance and resilience given repeated pulse disturbances (such as in a disturbance regime, e.g., fire or flooding), as well as press disturbances (Figure 4).

#### Growth rate

A tradeoff between growth rate and resource use efficiency (and hence competitive ability) may underlie the capacity of microbial populations to respond to disturbance (Stevenson and Schmidt, 2004). Enhanced growth rate is accompanied by a higher rate of synthesis of ribosomal components and is faster in microorganisms with more copies of rRNA-encoding genes, reducing the response time to favorable growth conditions for such organisms. When pulse disturbances are followed by favorable growth conditions, the fastest responders will multiply and alter community composition (Klappenbach et al., 2000), resulting in low resistance. By contrast, microorganisms with fewer copies of rRNA-encoding genes maximize efficiency of resource use (progeny per mole substrate; Lee et al., 2009), and may increase community resistance to press disturbances that result in long-term resource limitation. Genes other than rRNA genes distinguish bacteria optimized to grow at high nutrient (copiotrophic) and low nutrient (oligotrophic) conditions (Lauro et al., 2009). While the identity of rapid responders may be idiosyncratic across environments, quantifying the capacity of a microbial community for rapid growth or efficient resource utilization could inform hypotheses regarding each community’s compositional responses to pulse and press disturbances. Growth rate likely is important for microbial community resilience from pulse disturbances, as a few surviving individuals could grow quickly to recover to pre-disturbance population sizes after a sudden pulse, mortality-inducing disturbance, especially if the disturbance makes new resources available.

The relative growth rates of interacting microbial communities also can have implications for microbial resilience and resistance to press disturbances. A recent study compared the drought responses of a fungal-based food web in grassland soil with that of a bacterial-based food web in agricultural soil (de Vries et al., 2012). The results of this study suggested that relatively slower-growing fungi were more resistant to drought, but less resilient, while relatively faster-growing bacteria were less resistant but more resilient. The authors built structural models to assess the impact of fungal and bacterial drought responses on microarthropod (grazers of bacteria and fungi) richness, soil respiration, nitrogen dioxide production, and nitrogen leaching. This study demonstrated that press disturbances that alter microbial food webs may also influence soil resource availability. It also showed that the stability of microbial food webs was contingent, at least in part, on the ratio of slower-growing fungi to faster-growing bacteria.

#### Stochastic gene expression

Another means by which a microbial population can respond quickly to environmental change is through stochastic gene expression (Avery, 2006; Raj and van Oudenaarden, 2008), a process that samples multiple phenotypes and hence, like dormancy, is also considered a bet-hedging strategy. The presence of persister cells – dormant variants within a bacterial population that are tolerant to antibiotics (e.g., Lewis, 2010) – is one example of an alternative phenotype that increases fitness in an environment experiencing a transient selective pressure. Stochastic gene expression is among the multiple pathways that can lead to the formation of persisters (Lewis, 2010), a phenomenon that may have many parallels in non-pathogenic microorganisms. Stochastic gene expression may be key for microbial community resistance to pulse disturbances, as it offers a short-term strategy for survival of individuals that can re-populate a community after disturbance.

#### Dispersal and immigration

The large population sizes and rapid dispersal abilities (e.g., Finlay, 2002) of microorganisms can play an important role in community recovery after disturbance. Dispersal is a key feature of metacommunity theory, which recognizes communities as collections of interacting local communities linked by the movement of individuals in heterogeneous landscapes (e.g., Leibold et al., 2004; Logue et al., 2011). Disturbances initiate iterations of community re-assembly by killing or inactivating local resident taxa, releasing resources, and creating empty niches. These empty niches can be filled by local taxa (resistant taxa or taxa retrieved from seed banks) or by immigrants that arrive from other localities within a metacommunity.

Colonists dispersed from nearby localities (patches) can “re-seed” microbial populations that have become locally extinct after disturbance, thereby facilitating community resilience (Figure 4). For example, freshwater bacterial communities disturbed by a pulse salinity increase were both compositionally and functionally resilient because of continuous dispersal of microorganisms from an undisturbed source community (Baho et al., 2012). As another example, protozoan and rotifer population densities were more resilient after a recurring pulse disturbance (replacing 99% of a mesocosm’s contents with sterile media) when the disturbed mesocosm was connected to an undisturbed one (Altermatt et al., 2011a) than when the disturbed mesocosm had no refuge.

On the other hand, if disturbances are wide-spread such that they affect entire regions (e.g., climate effects, such as heat waves), dispersal could promote the dissemination of disturbance-tolerant taxa among localities (Eggers et al., 2012), thereby changing the dominant membership of a community and decreasing overall community resilience at both local and regional scales. For example, marine microalgae communities disturbed by a simulated heat wave were sensitive but not resilient because of a shift in community dominance toward a temperature-tolerant species. This species also became prevalent in other patches connected by dispersal, even though the conditions there were less suitable for it (Eggers et al., 2012).

Furthermore, niches opened by disturbance events are subject to stochastic colonization events by dispersed microorganisms. “*Priority effects*” refers to the impact that successful early colonizers may have on community re-assembly after disturbance, which can affect the likelihood of colonization by subsequently dispersed microorganisms (e.g., Shulman et al., 1983). Early post-disturbance colonizers that adapt rapidly to local conditions may persist long-term, out-competing native community members and impeding community resilience (Urban and De Meester, 2009).

Together these studies and others demonstrate that microbial dispersal, in interaction with biological attributes of local resident populations (plasticity, dormancy, or evolution) and disturbance characteristics, can have important implications for the resilience of microbial communities.

### Community properties: Diversity, turnover, and emergent properties

#### Diversity in all of its forms

In general, diversity is thought to influence how communities respond to disturbance. Aspects of alpha diversity, such as species richness and evenness, have been shown to enhance the functional resilience of communities of larger organisms (Allison, 2004; Downing and Leibold, 2010; Van Ruijven and Berendse, 2010), whereas evidence about the impact of richness and evenness on microbial community resilience is mixed (e.g., Griffiths et al., 2000a; Wertz et al., 2007; Wittebolle et al., 2009; van Elsas et al., 2012).

One challenge lies in clarifying the functional and compositional responses to disturbances of diverse communities. The underlying mechanisms behind a positive relationship between taxon diversity and resilience may be related to a buffering effect, called the *Insurance Hypothesis* (Yachi and Loreau, 1999). More genetically diverse communities are more likely to contain taxa with complementary response traits (e.g., Tilman et al., 1997, 2006; Lavorel and Garnier, 2002; Elmqvist et al., 2003) and the ability for rapid compensatory growth after a disturbance (e.g., Flöder et al., 2010), which may promote resilience. Also, rare microbial taxa (potentially below the limit of detection, and therefore not counted in estimates of community diversity) may quickly respond to altered environmental conditions and become abundant. This is exemplified by the case of a rare *Vibrio* species that was below detection in the majority of the time points over a 6-year study in the Western English Channel, but then bloomed to become a prevalent member of the community at one time point (Caporaso et al., 2011). Co-occurrence networks were used to find that the *Vibrio* bloom was correlated to a bloom of a diatom species (Gilbert et al., 2012). Though models are constantly improved for predicting conditions for microbial blooms (Larsen et al., 2012), niche spaces for many environmental microorganisms remain uncharacterized, which further veils the relationship between compositional and functional diversity and microbial community stability.

Here we discuss a few examples (of many in the literature) of the impact of diversity on microbial community stability. In an early experiment to tease apart the relations between diversity and community functions, Griffiths et al. (2000b) examined the impact of soil microbial diversity on functional stability using a range of intensities of soil fumigation followed by a disturbance, either a heat shock (pulse disturbance) or the addition of the heavy metal copper (II) sulfate (press disturbance). The results indicated that microbial production (rate of generation of biomass, here measured as thymidine incorporation) was not affected, but specific functions, such as nitrification, decreased when diversity was lower. The lower-diversity communities were less functionally resistant (measured as grass residue decomposition rate) to the press disturbance and unable to recover, but were sometimes more resistant to the pulse disturbance than the higher-diversity communities. The control community, which was not fumigated and had the highest diversity, was often the most resilient to both pulse and press disturbances.

Building on the Griffiths et al. (2000b) results, the relationship between species richness and stability was investigated for denitrifiers and nitrite oxidizers (Wertz et al., 2007), two specialized functional groups of soil microorganisms important for nitrogen cycling. In this work, microbial abundance (and, as an extension, richness) was altered by inoculating soil microcosms with different dilutions of microbial cells from non-sterile soil. After a period of incubation, the microcosms were subjected to a pulse heating disturbance. Denaturing gel gradient electrophoresis fingerprints of the denitrifiers and the nitrite oxidizers were coupled with measurements of the two processes, and each responded differently to heating. Though both processes were sensitive, denitrification was resilient after 3 months, while nitrite oxidation did not completely recover. The response of richness to heating was variable across dilutions, and richness did not recover to pre-disturbance levels after 3 months. These results suggest that a community may recover in function even if diversity remains altered after disturbance, and that the initial richness may not necessarily impact functional recovery.

A microcosm experiment was performed to specifically examine the role of initial community evenness (equitability of taxa abundances) on functional stability of denitrifiers subjected to a press increase in salinity and temperature (Wittebolle et al., 2009). The authors found that high initial evenness was important for functional stability of microbial communities. However, the communities were not observed after the disturbance ceased to assess resilience of function or evenness. This work demonstrated that aspects of diversity other than species richness can play a role for community functional stability. Given that many environmental microbial communities are characteristically uneven because they contain a large number of rare taxa (sometimes referred to as the “rare biosphere,” e.g., Casamayor et al., 2001; Sogin et al., 2006), the implications of small differences in evenness among generally uneven communities may be of interest for further investigation.

A very recent experiment demonstrated the impact of diversity on stability by creating synthetic combinations of soil isolates, rather than confronting the complexity and unknown organisms of the natural soil (van Elsas et al., 2012). With constructed mixtures containing various numbers of random representatives of a collection of cultured isolates, the authors demonstrated a highly significant correlation between species richness of the community and its resistance to invasion by an *E. coli* strain (van Elsas et al., 2012).

Together, these studies and others reveal the complex relationships between microbial community diversity, function, and stability. Multiple aspects of diversity (richness, evenness) can affect microbial functional resistance and resilience, and general and specific community functions may have different overall responses to pulse disturbance. Importantly, these studies suggest that there may not be a “one-size-fits-all” response of microbial diversity and function to disturbance. For diversity-stability relations, more work must be done to understand system-specific trends before it will be possible to determine which patterns, if any, are general across microbial systems.

#### Compositional turnover

Community turnover is the replacement and substitution of community members along an environmental gradient or with time (Wilson and Shmida, 1984). Turnover is directed by the growth of populations, and partially determines how quickly a community recovers from a pulse disturbance (Figure 4). High-throughput fingerprinting and sequencing tools that enable experiments involving longer time series demonstrate that many microbial communities have clear trajectories. For example, successional patterns have been observed in tree leaf bacterial communities over the growing season (Redford and Fierer, 2009; Redford et al., 2010), in the human gut after antibiotic treatment (Antonopoulos et al., 2009; Dethlefsen and Relman, 2011), and in the infant gut during the first two and a half years of development (Koenig et al., 2011). Also, seasonal trajectories are common in freshwater, marine, and sediment systems (Fuhrman et al., 2006; Christian and Lind, 2007; Shade et al., 2007; Nelson, 2008; Andersson et al., 2009; Crump et al., 2009; Gilbert et al., 2009, 2010). Together these studies provide insight into the temporal scale on which community turnover and disturbance responses may be anticipated in similar systems, but turnover is unknown for many other habitats. Further investigation is needed to describe, quantify, and compare turnover within and across habitats.

Species-time relationships, which quantify the accumulation of new species in a community with time, provide a method to calculate community turnover (White et al., 2006). There is preliminary evidence that species-time relationships of community turnover may be common among microbial communities from very different environments. For example, microbial communities in streams (Portillo et al., 2012), on leaf surfaces (Redford et al., 2010), across a set of newly deglaciated soils (Nemergut et al., 2007), and in bioreactors (Van Der Gast et al., 2008), have exhibited species-time relationships with turnover rates comparable to those of larger organisms. If most microbial communities display a characteristic turnover rate, as observed for communities of larger organisms (White et al., 2006), variation around the expected range of community turnover rates may be a reasonable starting point for comparing microbial community responses to disturbance.

Similarity-decay (Nekola and White, 1999) is a common method for understanding changes in microbial community structure over space (e.g., Horner-Devine et al., 2004; Jones et al., 2012), but has also been applied over time in communities of larger organisms (Korhonen et al., 2010). For temporal similarity-decay, all pairs of community resemblance (*a.k.a*. similarity or distance) are regressed against time (or space) between community observations. The slope of this regression is analogous to a turnover rate, but has additional utility from species-time relationships because the resemblance metric can be chosen to include properties of community composition (e.g., Sørensen), structure (e.g., Bray–Curtis), and phylogenetic representation (e.g., UniFrac distance, based on the underlying calculation of phylogenetic diversity, Faith, 1992). Similarity-decay has been applied to understand temporal dynamics of microbial communities (e.g., Wittebolle et al., 2008; Bürgmann et al., 2011). As one example, similarity-decay was used to compare resilience of lake microbial communities across treatments in an experiment designed to separate the environmental drivers of oxygen and nutrients from the physical process of water column mixing, an important seasonal disturbance to temperate lake bacterial communities (Shade et al., 2011). In this study, similarity-decay relationships were used in part to quantify the relative robustness of the communities to these environmental disturbances, such that the hypolimnion community was found to be the most sensitive to oxygen addition, but also the most resilient. Though similarity-decay clearly is useful as a baseline descriptor of temporal community turnover, this study demonstrated the additional utility of similarity-decay for comparing microbial community resilience when challenged with different disturbances.

#### Emergent properties: microbial communities as networks

Interactions between community members, including competition and mutualism (Little et al., 2008), are also important in determining community response to disturbances. For example, a mesocosm experiment with protist communities demonstrated that competitive interactions and disturbance characteristics together determined the community disturbance response, where competition between species became increasingly important in driving extinction as the intensity of disturbance was amplified (Violle et al., 2010). This trend was observed despite the fact that the species were chosen for the experiment because they collectively represented a wide range of competitive ability and disturbance tolerance. Thus, the community response was more complex than the sum of the individual species’ traits, and instead hinged on interspecific interactions.

As time series analyses of microbial communities become increasingly available, we will be better able to quantify complex interactions among community members. For instance, recently developed statistical tools enable investigation of microbial interactions by studying co-occurrences of microbial taxa through time. Networks can be built from co-occurrences, with nodes representing taxa or operational taxonomic units (OTUs) and connecting edges representing correlation over time (e.g., Ruan et al., 2006). These networks assess effects of disturbance on community dynamics (Montoya et al., 2006). Positive co-occurrence may indicate common preferred environmental conditions or cooperative activities (facilitation and/or syntrophy). Similarly, negative correlations may represent the outcome of competition (i.e., displacement) or negative interactions, such as allelopathy or predation (Fuhrman, 2009). Co-occurrence networks are gaining popularity in microbial ecology (Faust and Raes, 2012), and recently have been applied to observational studies of marine and soil systems (Steele et al., 2011; Barberan et al., 2012; Eiler et al., 2012; Gilbert et al., 2012). Network analysis applied to controlled experiments can provide insight into the associations between community members that are lost, gained, or maintained after a disturbance (Shade et al., 2010). Networks can also be used to discover “core” community members shared across communities from similar habitats (Shade and Handelsman, 2012), serving as a method to identify taxa shared across localities within a larger metacommunity.

Theoretical and empirical work on food webs indicate that the robustness of such networks is affected by several attributes, such as the strength of links among species (Montoya et al., 2006). In particular, architectural properties of trophic networks affect the relationship between network complexity and stability (May, 1974; Pimm, 1984; McCann et al., 1998; Rozdilsky and Stone, 2001). For example, in mutualistic networks, compositional diversity and connectivity lead to higher resilience, whereas these properties destabilize trophic networks (Thébault and Fontaine, 2010). Microbial communities are probably affected by both mutualistic and trophic interactions, and thus may provide a unique system for exploring the relationship between network attributes and community stability. Though we hypothesize that microbial interactions and the emergent network properties of a microbial community likely contribute to both resistance and resilience to pulse and press disturbances (Figure 4), the exact mechanisms underlying this stability often are unknown, and may be challenging to unravel for many species-rich microbial communities that maintain both mutualistic and trophic interactions among their members.

## Outlook: Communities as Systems of Genes and their Functions

Microbial ecology is in a unique position in the larger field of ecology. Since the inception of community ecology, studies of the nature of communities at all taxonomic levels have been challenged by common difficulties. Producing a complete census, controlling variables, sampling completeness, and accounting for low abundance members are typical problems that have confronted all community ecologists, including microbial ecologists. However, the advent of meta-omics has provided microbiologists with the tools to address each of these challenges in new ways (Gilbert et al., 2010; Teeling et al., 2012). Portraits of a community’s genes, gene expression, and metabolite production can be represented in a single sample, providing insight into system-level stability. Consequently, microbial ecologists are in a position to elucidate global principles in a manner that is not easily available in the broader field of ecology.

For example, time series of 16S rRNA, shotgun metagenomics, meta-transcriptomics, and meta-metabolomics data will allow researchers to quantify the number of functions shared across taxa from the same community, identify the taxa that are expressing genes for those functions at a given time point, and determine the functional output (in terms of number, abundance, and composition of molecules) from those transcripts. Analyzing this suite of information through time and in response to disturbances will provide quantitative insight as to how often and under what scenarios microbial community structure and function are linked, and whether those linkages are relevant for ecosystem processes. Applying this suite of tools to carefully designed disturbance experiments will additionally help to unravel mechanisms of community stability into different habitats. It will also provide key insights into defining ecologically relevant taxonomic and functional units for microorganisms. Thus, with information-rich datasets, precisely collected time series, and thoughtfully designed experiments (Knight et al., 2012), microbial ecologists are poised to test fundamental hypotheses in ecology, and to move forward in predicting stability of microbial communities in the face of novel disturbances.

## Conflict of Interest Statement

The authors declare that the research was conducted in the absence of any commercial or financial relationships that could be construed as a potential conflict of interest.

## Supplementary Material

The Supplementary Material for this article can be found online at http://www.frontiersin.org/Fractal_Physiology/10.3389/fphys.2012.00417/abstract

## Appendix

### Criteria for the literature analysis

To understand what is known about microbial community resilience, we conducted a review of the literature. Studies were searched using ISI’s Web of Knowledge with the following search strings: perturb*, fluctuat*, disturb*, robust*, resilien*, resist* AND “community structure OR diversity OR species composition” AND microb*, bact*. Studies were considered if they adhered to the following criteria: (1) explicitly addressed communities (including all aspects of diversity). Papers dealing with single species only were not included; (2) contained empirical data, either from observations or experiments. Conceptual or review papers were excluded.

Papers meeting these criteria were categorized as experimental (manipulation of environmental conditions) or observational (documented responses to naturally occurring disturbances). Information was recorded regarding study organisms, habitat, study duration, sampling frequency, community profiling method used, experimental setup, and dispersal limitations, and classification of the disturbance as press (stressor did not return to pre-disturbance conditions) or pulse (stressor ceased to pre-disturbance conditions). Results were summarized as the presence or absence of changes in community composition or function, and whether there was recovery to the pre-disturbance state. If available, we also noted the post-disturbance recovery time (resilience).

The literature search (18th of January 2012) yielded 3,312 unique references. 352 references were obtained after filtering according to the three primary criteria, and useful data could be extracted from 247 publications. Experimental studies included 310 entries in 196 publications, while observational studies included 68 entries in 51 publications. See Figure A1 for a detailed comparison of disturbance effects on community composition and function in different habitats and press and pulse disturbance broken up by different disturbance types.

Approximately eighty percent of the investigations included measures of microbial function, such as respiration, biomass production, or the activity of extracellular enzymes. The effects of dispersal on resistance and resilience were only rarely explicitly addressed (Altermatt et al., 2011b; Baho et al., 2012), however approximately one third of the studies used open systems or did not exclude dispersal. Study durations varied greatly, ranging from less than 1 day (Cleveland et al., 2007) to decades (Spiegelberger et al., 2006). In contrast to the rather short-term duration of experimental studies (median: 70 days), observational studies focused on long-term effects (median: 645 days). However, in both cases there was a significant negative relationship between experimental duration and sampling frequency (Figure A2), reflecting an astonishing congruency of expected effect sizes of resistance and resilience dynamics in microbial communities.
